# Hosting and managing large sets of virtual microscopy slides on the internet for E-learning and for reference

**DOI:** 10.1186/1746-1596-8-S1-S19

**Published:** 2013-09-30

**Authors:** Hans-Peter Sinn

**Affiliations:** 1 Department of Pathology, University of Heidelberg, 69120 Heidelberg, Germany

## Background

While the imaging technology for virtual microscopy can be considered mature, the techniques used for the organization and presentation of the digital slides are more complex because of the diversity of user requirements for every project and the technical challenges regarding the structuring and management of the slides. Generally, the presentation of virtual microscopy on the intra- and internet requires the availability of tools that act as a bridge between the digital slide repository and a content management system (CMS) for the web presentation. This involves specific programming of the CMS in order to display the virtual slides in a consistent and specific manner and provide for easy expandability. This is true for E-Learning systems, but also for general digital slide repositories on the web. We have created internet platforms for undergraduate and postgraduate pathology teaching and reference and will present our approach for an effective management and presentation of large numbers of digital slides.

## Materials and methods

Educational slides were digitized using an Aperio CS scanner and stored in the Aperio svs file format, using JPEG and JPEG2000 compression. The Aperio Spectrum platform was used as the primary image and metadata repository, but because not used to publicly display its content because of its technical limitations and licensing issues. Therefore, all images and metadata information were exported to specialized E-Learning and pathology reference platforms. The implementation of digital pathology slides on these platforms included the following common important aspects:

1. Load balancing: The simultaneous access of up to 70 students in the classroom to few digital slides at one time, or dozens of users to many slides on an open internet platform requires effective load balancing for the image server. This was achieved by setting up a reverse proxy load balancer on Apache that distributes the load to several image server processes which are running on subordinated machines.

2. For E-Learning, the Moodle open source E-Learning platform was chosen, making use of its database module and customized javascripts. With this approach, a generic format for the presentation of the slides was created that allows for adding structured metadata, side-by side display of normal and pathological slides, and multislide images of the same entity in various special stains. Thumbnail images that are provided by the image server software are embedded dynamically.

3. For the postgraduate digital slide pathology repository, a general purpose content management system (CMS) was chosen (Textpattern), that builds upon the open source LAMP (Linux, Apache, MySQL, PHP) architecture. All metadata, and including the links to the virtual slides could be seamlessly transferred from the Spectrum database into Textpattern.

4. The Textpattern CMS was programmed to provide for a hierarchical structure that reflects the pathological disease taxonomy in a hierarchical fashion. It uses a tree structure with unlimited depth, and is easily expandable. Digital slides are assigned to one or more categories in this system.

## Results and discussion

The user of a pathology reference site expects a systematic layout of the internet site, allowing for easy access of its content based on the medical disease categories, and to be able to browse related entities more easily than in a medical textbook. Because of the many synonyms and related terms of medical terminology, it is not sufficient to rely on a built-in search engine to access the digital slides in question. For an effective approach, a nosological hierarchy of the digital slides must be built that is governed by the pathological diagnosis and organ sites in focus. This requires the building of hierarchical and associative relationships for the diagnostic terminology as well as the use of dictionaries and thesauri. In this fashion, the content management system is able to focus on the pathological slides searched for, and at the same time provide links to related entities in the database. An associative and disease-oriented browsing becomes possible.

With undergraduate and postgraduate pathology teaching, the focus is on the display of cases series that illustrate a pathologic process or the topic of a seminar or a diagnostic entity. Here, the impact is on the linking of the pathology slides with metadata and the display of related slides, such as special stains, differential diagnoses, or normal counterparts. Therefore, a format must be chosen that allows for the consistent building and management of cases series with structured metadata, such as clinical or disease related information, related slides, and other data, such as PDF files. E-Learning systems such as Moodle that provide modular plugins for multimedia data can be used for this purpose. The linking the the digital slides with the content on the E-Learning system, and the handling of different browser requirements can be achieved by custom Javascript programming in the E-Learning system.

Both pathology reference sites and E-Learning sites which present digital slides must have sufficient server capacity to be able to deal with multiple concurrent users who are accessing the same or different digital slides simultaneously. A typical example is a class of more than 50 medical students that use virtual microscopy in the classroom. The user experience of quickly navigating the digital slides is dependent on an effective load balancing solution and an image server technology that supports this. Today’s advanced servers with multiprocessor and multicore architectures allow for the upscaling of digital pathology proportional to the number of cores in one machine, and thus limit the need of running multiple servers in parallel for the handling of the image data.

## Conclusion

The building and management of a digital pathology internet site for the purpose of pathology teaching and reference requires special attention to the database design, the handling of metadata, the user interface, and load balancing.

## Competing interests

The author declares no competing interests.

## Authors’ contributions

H-PS designed the study, contributed the experimental data, and wrote the manuscript.

